# Sclerosing Epithelioid Fibrosarcoma of the Spine: Diagnosis and Treatment of a Rare Entity

**DOI:** 10.7759/cureus.42143

**Published:** 2023-07-19

**Authors:** João Lima, Andreia Coutada, Mariana Afonso, Artur Aguiar, Mavilde Arantes

**Affiliations:** 1 Radiation Oncology, Portuguese Oncology Institute of Porto, Porto, PRT; 2 Pathological Anatomy, Portuguese Oncology Institute of Porto, Porto, PRT; 3 Neuroradiology, Portuguese Oncology Institute of Porto, Porto, PRT

**Keywords:** magnetic resonance (mr), lumbar spine tumor, neurosurgery oncology, external beam radiation therapy, sclerosing epithelioid fibrosarcoma

## Abstract

Sclerosing epithelioid fibrosarcoma (SEF) is a rare subtype of sarcoma with high rates of local recurrence and distant metastasis. Morphologically, it resembles other mesenchymal and non-mesenchymal tumors, making it a diagnostic challenge. Treatment relies mostly on surgery with adjuvant chemotherapy or radiotherapy (RT).

A 46-year-old woman who presented with lumbar pain and weight loss underwent a computed tomography (CT) scan, magnetic resonance imaging (MRI), and a [18F]-fluorodeoxyglucose positron emission tomography-computed tomography (18F-FDG PET/CT) scan, which showed a lesion involving the L5 vertebra. An incisional biopsy of the lesion established the diagnosis of SEF, with diffuse expression of MUC4 and focal expression of EMA.

The patient was treated with neoadjuvant RT followed by surgery. Histology was congruent with the previous diagnosis and demonstrated post-radiation changes.

In conclusion, SEF is an aggressive type of sarcoma that is easily misdiagnosed, so it is important to consider it in the differential diagnosis to avoid unbeneficial treatments and a detriment to patient survival.

## Introduction

Sclerosing epithelioid fibrosarcoma (SEF) was first described by Meis-Kindblom et al. in 1995 as a rare and distinctive subtype of sarcoma with fibroblastic differentiation and unique architectural features [1-5]. SEF most commonly arises in the deep musculature, but it can also originate in the bone, with the spine being a rare primary site for SEF [1,3,6]. It is recognized as a fully malignant tumor with high local recurrence rates and distant metastasis, despite its relatively innocuous morphology [1,2,7]. Its typical histological features include nests and cords of small, uniform monomorphic epithelioid cells embedded in a densely sclerotic matrix that is indistinguishable from osteoid. Its cells classically present light eosinophilic to vacuolated cytoplasm and round nuclei with fine chromatin [6,8]. The diagnosis of spinal SEF is challenging, not only because of its rarity but also due to its morphological similarities with other mesenchymal and non-mesenchymal tumors [1].

In the last decade, the ability to diagnose SEF accurately has further improved, especially due to the strong cytoplasmic immunohistochemical expression of mucin 4 (MUC4), which is highly sensitive and specific for SEF [1,8]. On a molecular level, the most common mutation found in SEF is EWSR1-CREB3L1 [4,6,8]. On computed tomography (CT), bone SEF presents as a lytic expansile lesion, mostly surrounded by sclerotic rims. Magnetic resonance imaging (MRI) usually shows a hypointense lesion both on T1WI and T2WI, with peripheral contrast enhancement [4,6,9]. Treatment for SEF consists of complete surgical removal of the lesion, with many surgeons preferring an en-bloc resection since it has been shown to reduce local recurrences. Usually, surgery is followed by chemotherapy or RT to improve local control [1,3,10].

This report is about the case of a middle-aged woman diagnosed with SEF involving the body of the L5 vertebra and treated in our institution. 

This article was previously presented as a meeting abstract at the 2023 Oncology Spring Meeting, held in Portugal from the 23rd to the 25th of March, 2023.

## Case presentation

**Figure 1:**
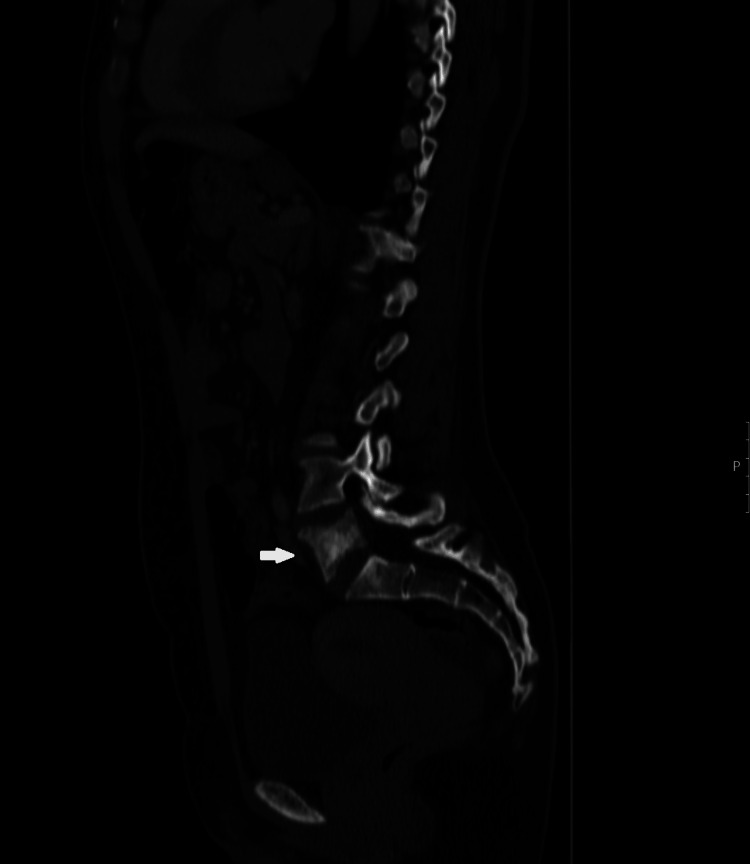
Lumbar spine CT scan, sagittal plane. A lumbar spine CT showed a lesion involving the body, left pedicle, and left superior articular facet of the L5 vertebrae.

**Figure 2:**
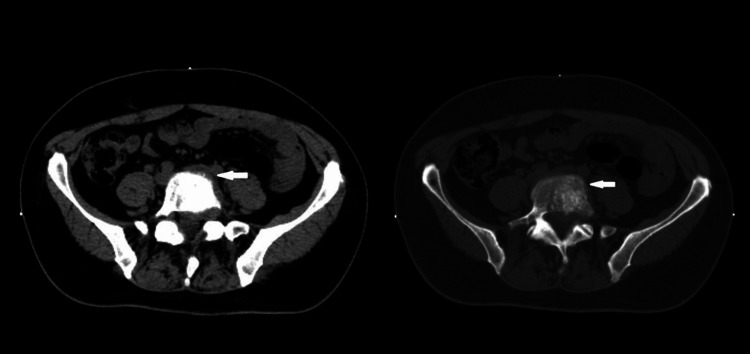
Lumbar spine CT scan, axial plane. A lumbar spine CT showed a lesion involving the body, left pedicle, and left superior articular facet of the L5 vertebrae.

**Figure 3:**
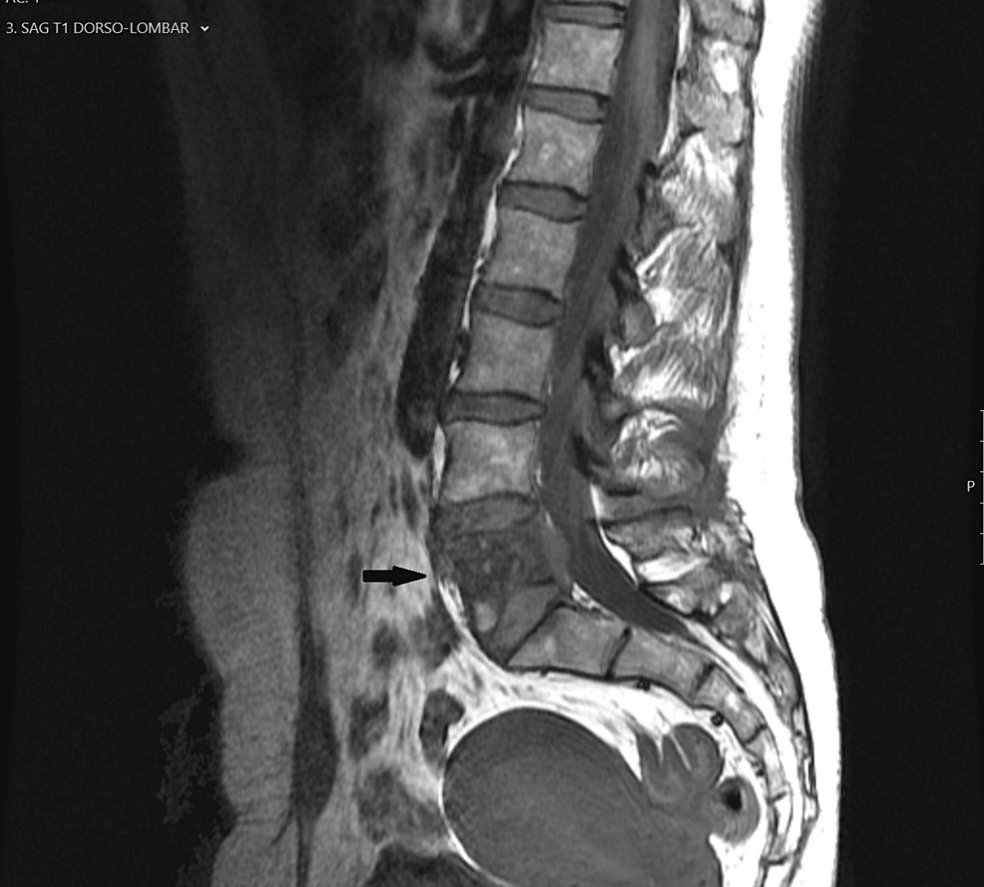
MRI, T1WI sequence. On MRI, the lesion was isointense on T1WI.

**Figure 4:**
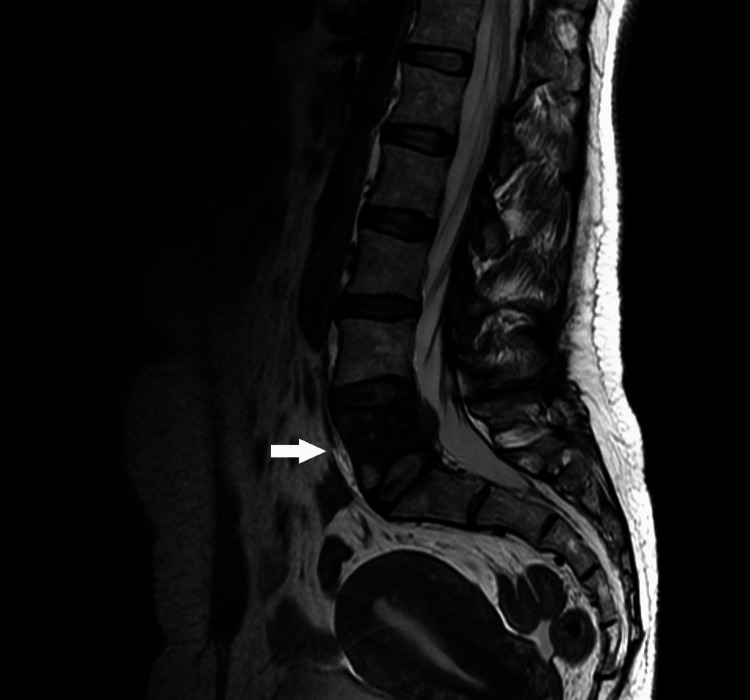
MRI, T2WI sequence. On MRI, the lesion was isointense on T2WI.

**Figure 5:**
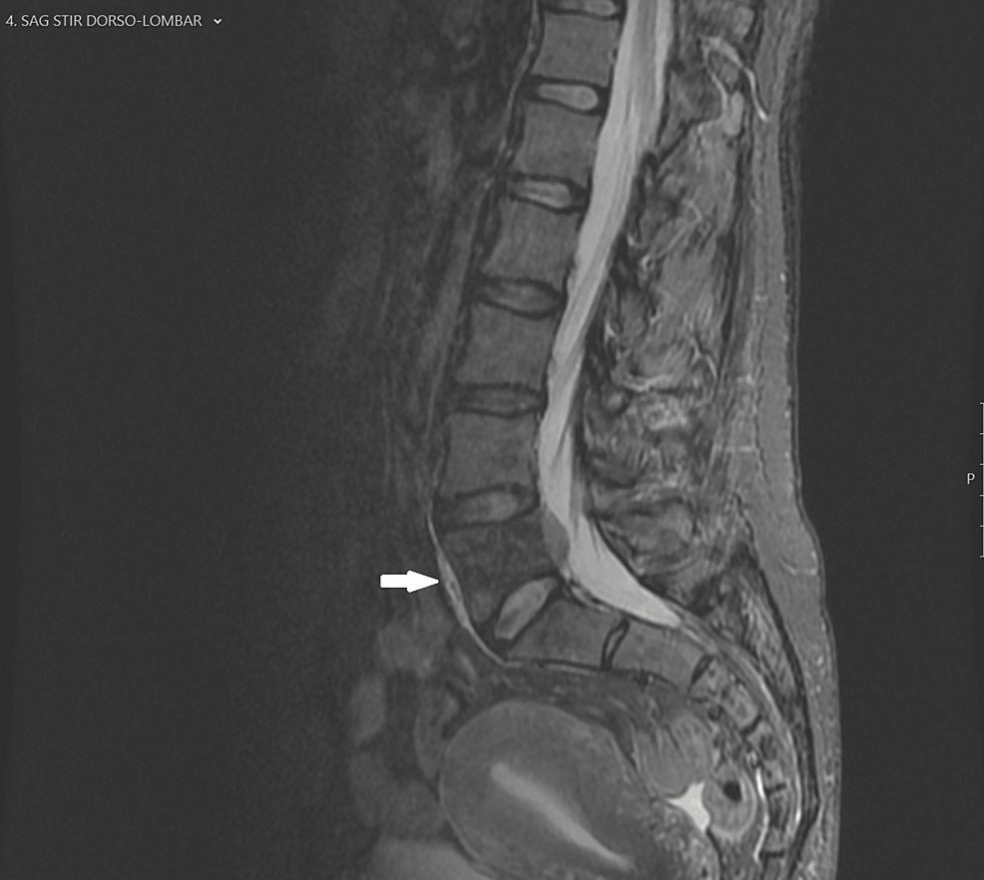
MRI, STIR sequence. On MRI, the lesion had no evident hypersignal on STIR sequences.

**Figure 6:**
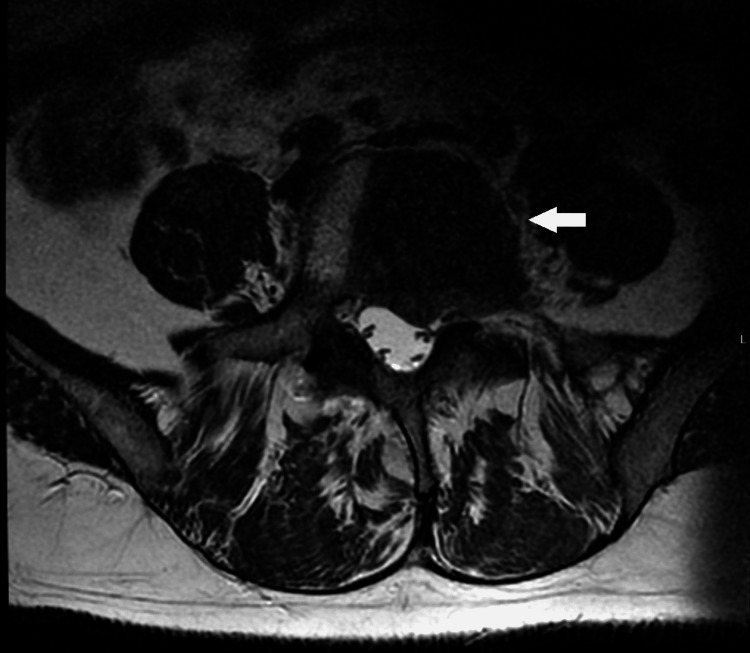
MRI, axial T2WI sequence. Axial T2WI highlights the existence of a left paravertebral soft tissue mass at L5, obliterating the left anterolateral aspect of the epidural space and the left L5-S1 foramen, probably with left L5 nerve root compression.

**Figure 7:**
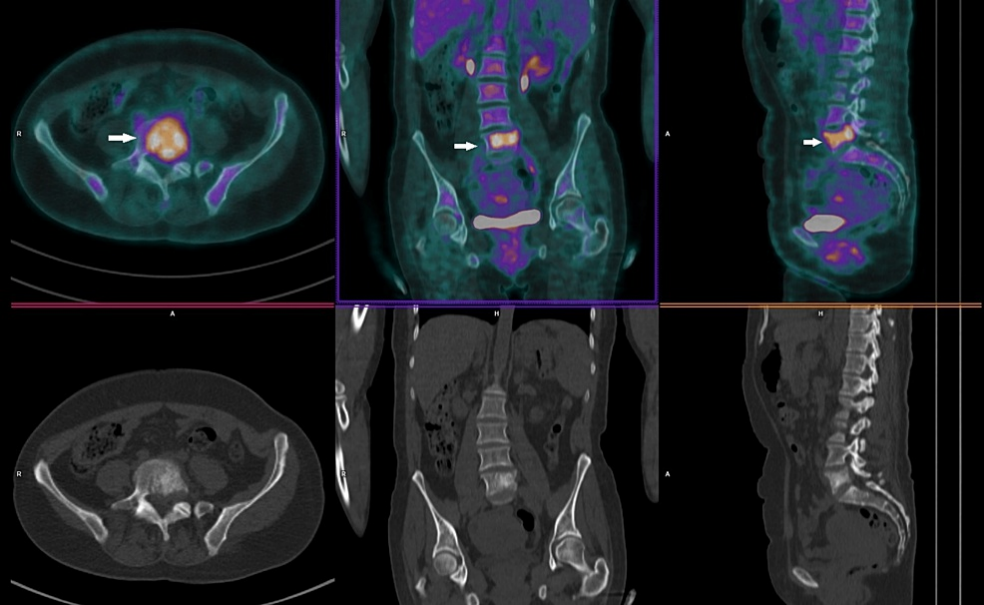
Staging 18F-FDG PET/CT scan. 18F-FDG PET/CT scan showing a hypermetabolic malignant lesion involving the body and left pedicle of the L5 vertebrae, with endocanal (left anterolateral) and foraminal (left conjugation hole L5-S1) insinuation related to the lesion described. There can also be seen a pathological fracture of this vertebral element. The remaining study did not show any other foci of anomalous radiopharmaceutical uptake that could suggest other hypermetabolic lesions.

The patient is a Caucasian 46-year-old woman, referred to our institution in September 2020, with a one-year duration of lumbar pain irradiating to the left lower limb, associated with > 10% weight loss in eight months. The dorsolumbar palpation did not reveal any abnormalities or cause any local pain. On neurological examination, Lasègue's sign was positive, and left foot extension was limited. In this context, a CT scan was performed, which revealed a lesion of 28.3 millimeters involving the body, left pedicle, and left superior articular facet of the L5 vertebra (Figures 1, 2). On magnetic resonance imaging (MRI), the lesion was hypointense on T1WI and T2WI, with no signal change on T2-weighted short-tau inversion recovery (STIR) and with abnormal contrast enhancement (Figures 3-6). The lesion was associated with a left paravertebral soft tissue mass with endocanalar and foraminal expression, which obliterated the left anterolateral aspect of the epidural space and the left foramen L5-S1, probably with left L5 nerve root compression. To exclude distant metastasis, the patient was also evaluated with a [18F]-fluorodeoxyglucose positron emission tomography-computed tomography (18F-FDG PET/CT) scan that demonstrated a hypermetabolic malignant lesion involving the left side of L5 (Figure 7).

**Figure 8:**
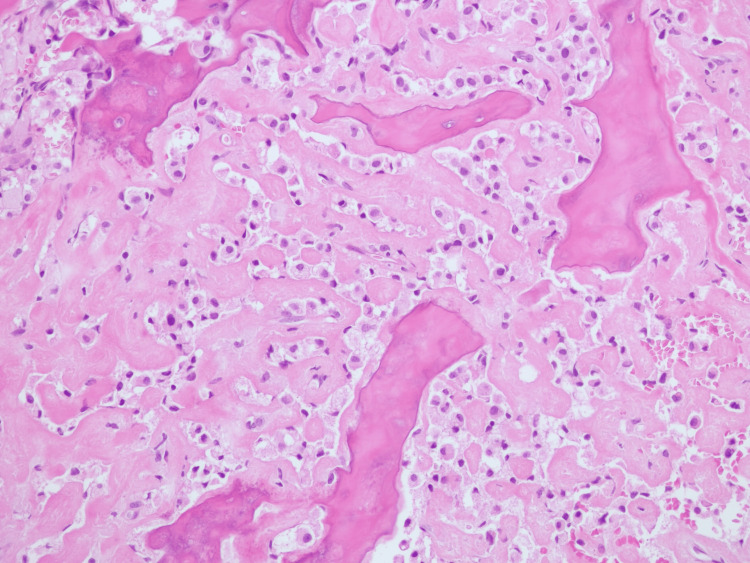
Histological study through incisional biopsy, showing haematoxylin and eosin (H&E) staining. Histological study through incisional biopsy showed on H&E staining a classic SEF morphology with polyhedric monomorphic cells with broad and pale cytoplasm and an ovoid nucleus, embedded in an abundant fibrocollagenous stroma with trabecular rearrangement.

**Figure 9:**
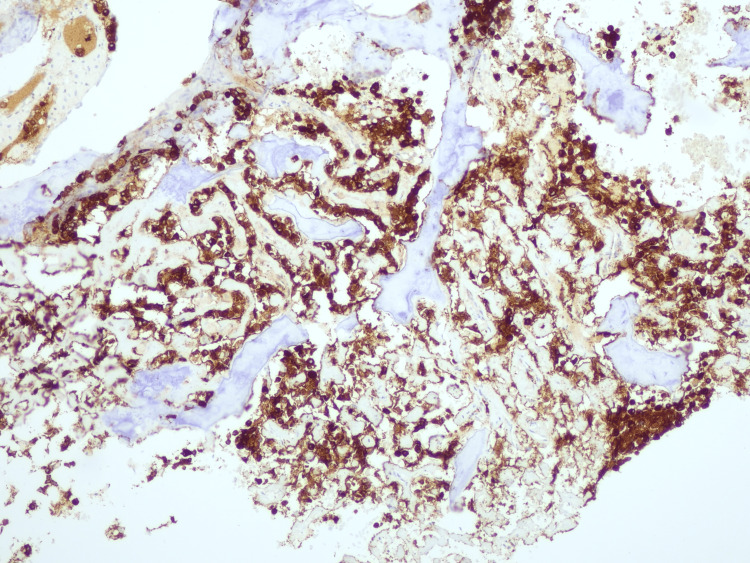
Histological study through incisional biopsy and immunohistochemical assay. Immunohistochemical assays show strong and diffuse expression of MUC4.

Given that a diagnosis including histologic subtype and grade is almost always necessary for the optimal treatment of a soft tissue sarcoma, a biopsy of the mass was necessary. Therefore, in November 2020, a percutaneous biopsy, guided by fluoroscopy, established the diagnosis of sclerosing epithelioid fibrosarcoma with diffuse expression of MUC4 and focal expression of EMA in the absence of expression of CK8/18, CK19, CD38, CD138, MUM 1, CD68, S100, SOX 10, C-Kit, and SATB2 (Figures 8, 9). Samples were sent for genetic testing by fluorescent in situ hybridization (FISH), which was unsuccessful in providing any information due to the physical characteristics of the sample.

**Figure 10:**
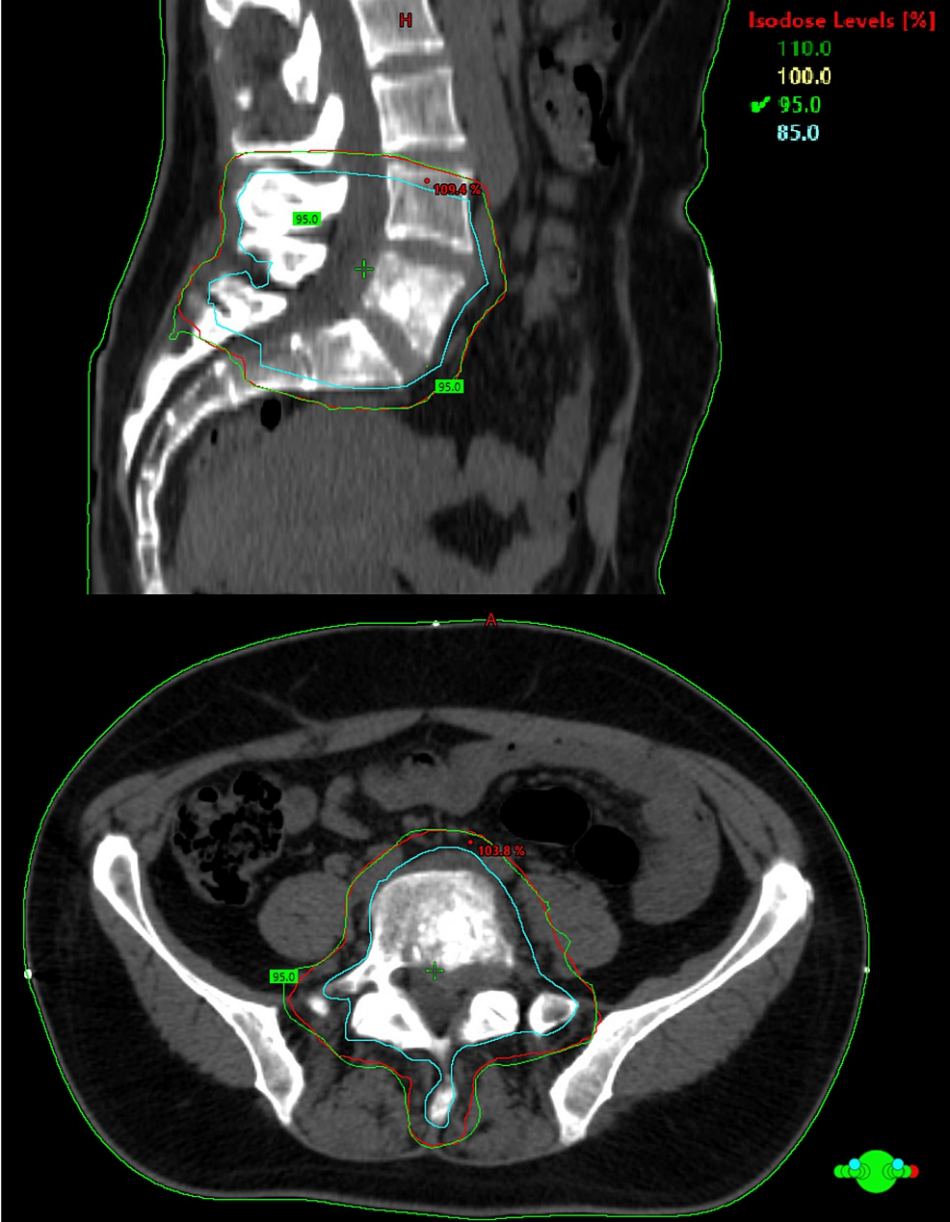
RT Plan showing CTV (blue) and PTV (red) and the 95% isodose line.

**Figure 11:**
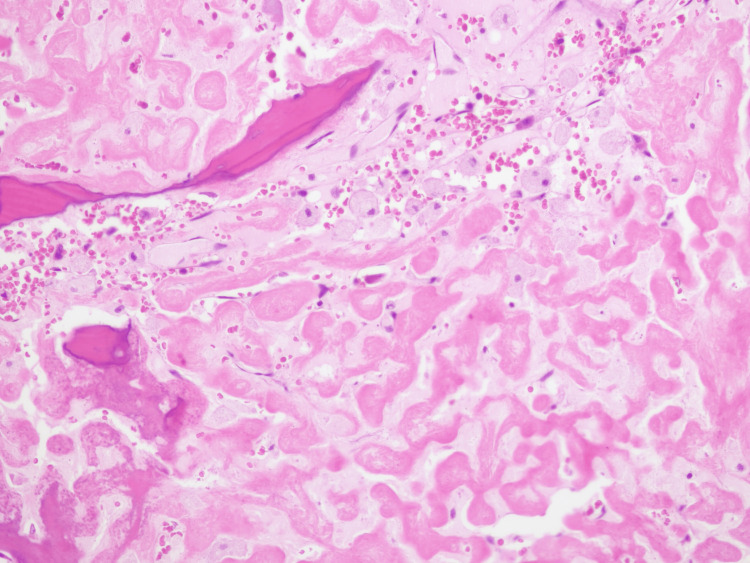
Histological study through excisional biopsy. Excisional biopsy, post-RT, presents with morphological aspects partially overlapping with those observed previously, with alterations induced by the previous therapy performed; fragments of the pulposus nucleus are also identified.

**Figure 12:**
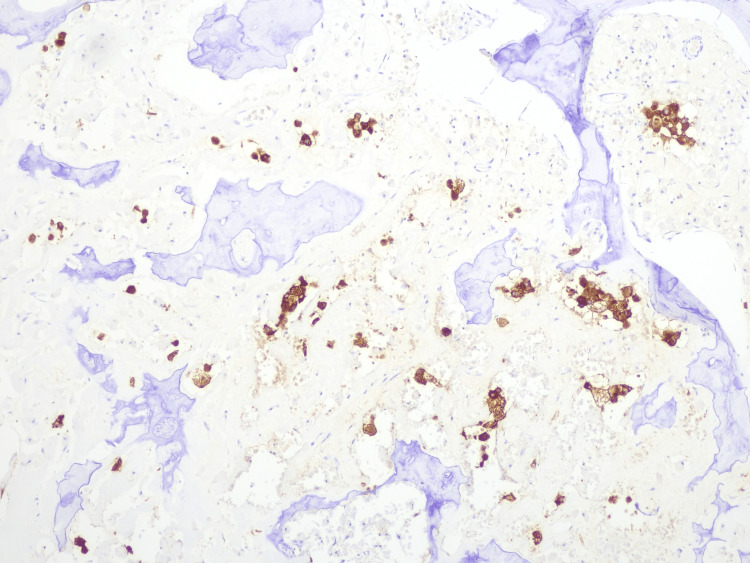
Histological study through excisional biopsy and immunohistochemical study. The correspondent immunohistochemical study performed documents diffuse positivity for MUC4.

**Figure 13:**
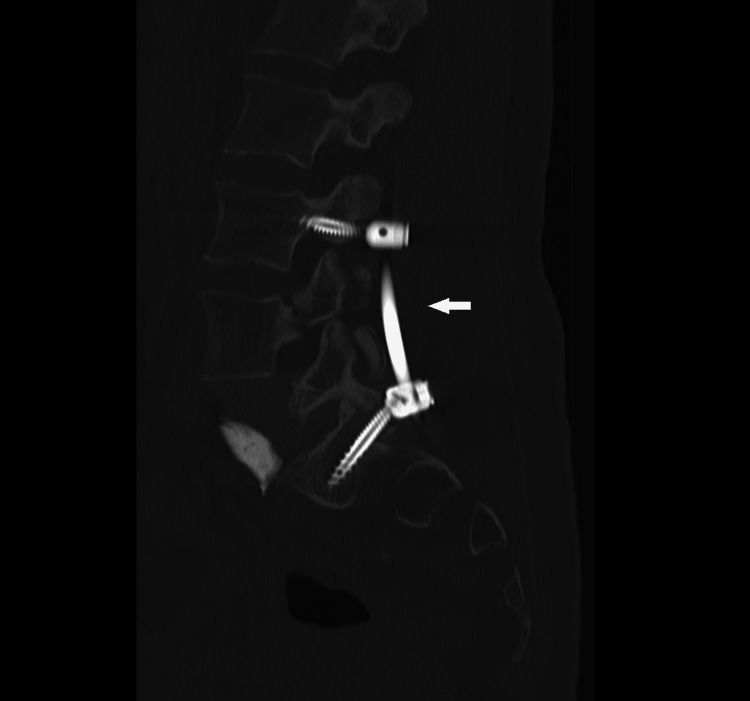
Lumbar spine CT scan after surgical posterior stabilization L3-S1.

**Figure 14:**
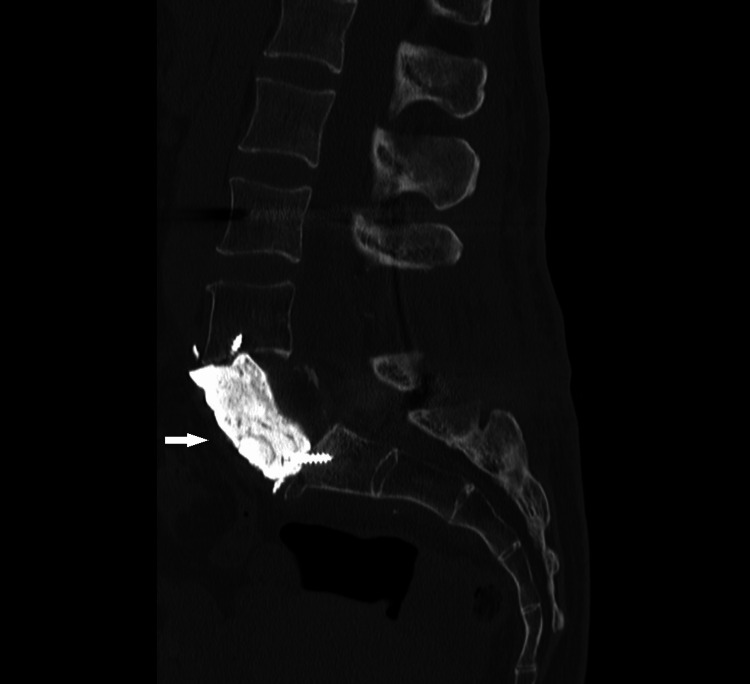
Lumbar spine CT scan after ventral corpectomy with reconstruction of the defect with cementoplasty.

**Figure 15:**
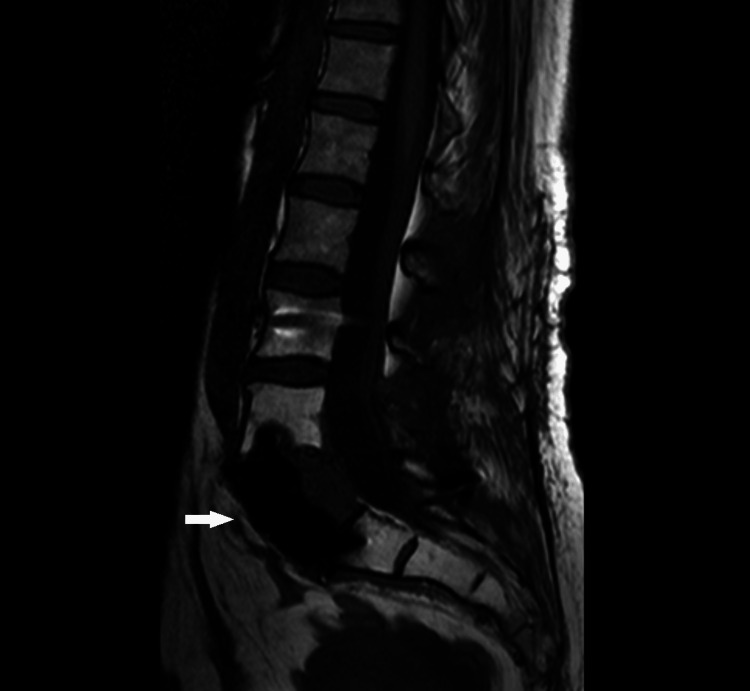
Lumbar MRI, sagittal T1WI sequence. Lumbar MRI after surgery showed no persistence of disease on sagittal T1WI.

**Figure 16:**
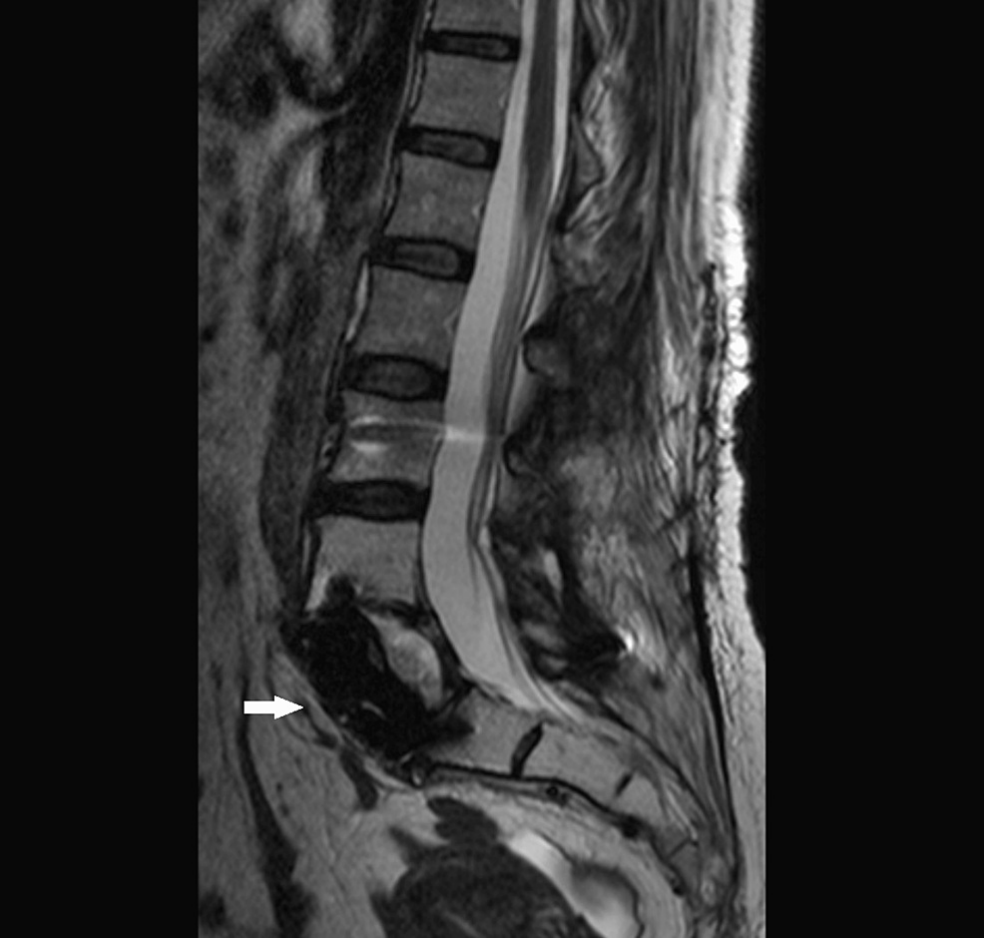
Lumbar MRI after surgery, sagittal T2WI sequence. Lumbar MRI after surgery showed no persistence of disease on sagittal T2WI.

**Figure 17:**
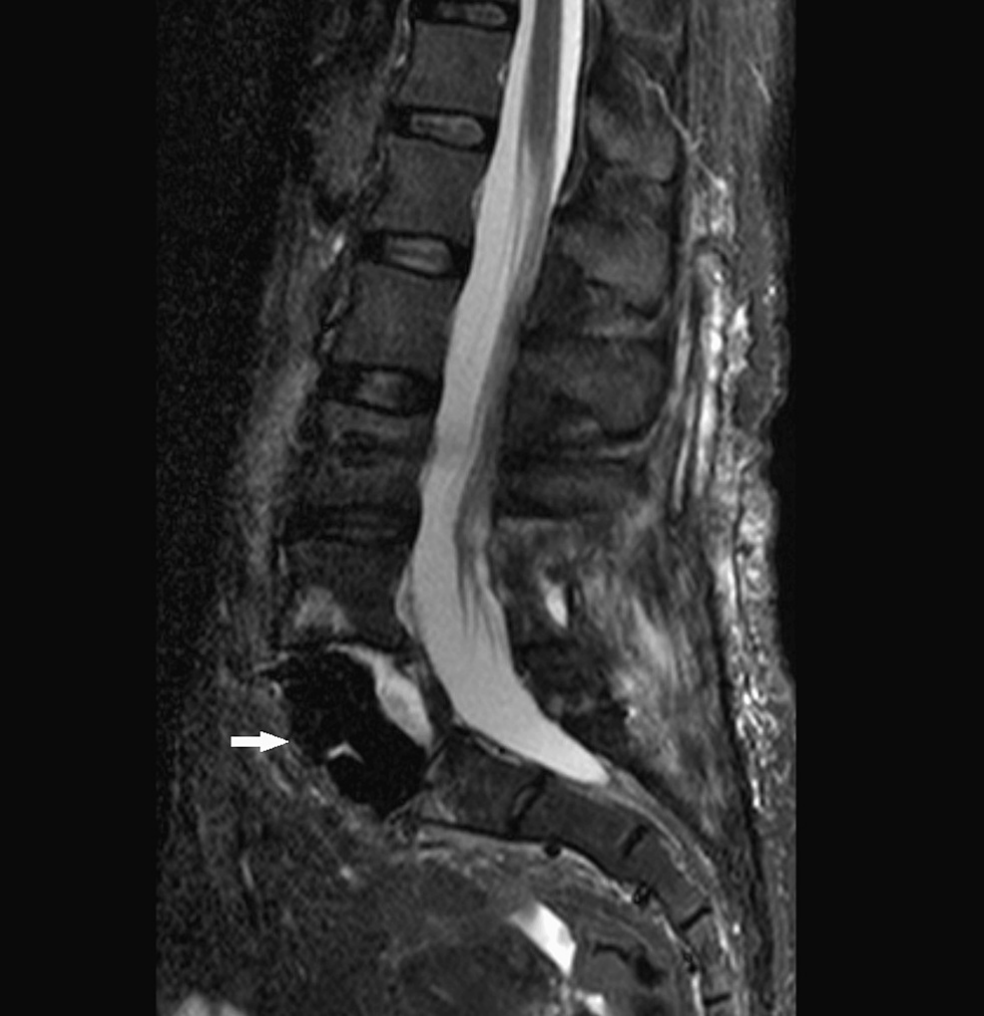
Lumbar MRI after surgery, sagittal T2-STIR. Lumbar MRI showed no persistence of disease on sagittal T2-STIR.

**Figure 18:**
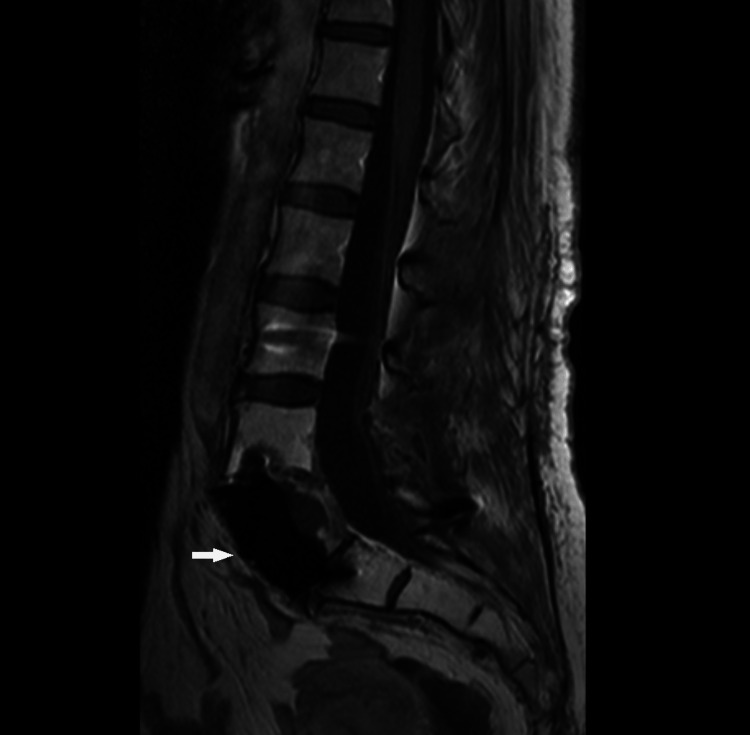
Lumbar MRI after surgery, sagittal enhanced T1WI. Lumbar MRI showed no persistence of disease on sagittal-enhanced T1WI.

**Figure 19:**
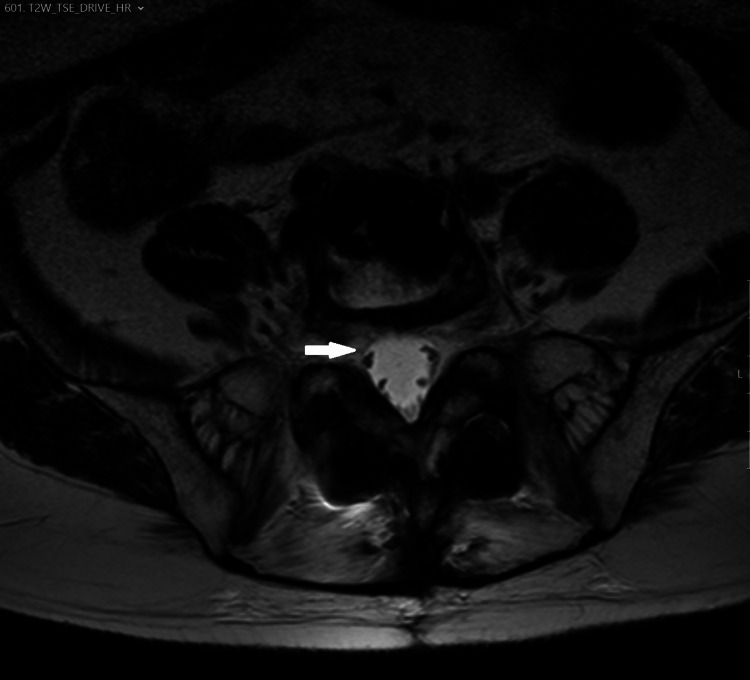
MRI after surgery, axial T2WI. Axial T2WI showed no soft tissue mass on the vertebral canal or foramina at this level. The left L5 nerve root appeared normal.

Since there were no other areas of involvement identified on the PET/CT, the lesion at L5 was considered a primary bone neoplasm. The multidisciplinary team decided to treat it with neoadjuvant RT followed by surgery, given the unlikely benefit of systemic treatment. An initial decompression surgical procedure was avoided due to the high risk of tumor seeding. Furthermore, a total dose of 50 Gy to the spine was safer than the doses used in adjuvant RT (60-70 Gy), considering the proximity to the cauda equina and the nerve roots. After CT simulation, GTV (gross tumor volume) was delineated to encompass the entire L5 vertebra, including the soft tissue component of the tumor; a CTV (clinical target volume) expansion of 2 cm was performed superiorly and inferiorly, encompassing adjacent bone structures; and PTV (planning target volume) was obtained by extending an 8 mm isometric margin from CTV. RT was delivered using IMRT for a total dose of 50 Gy, split into 25 fractions of 2 Gy per day, from the 3rd of May 2021 to the 4th of June 2021, with no interruptions (Figure 10). Given the anatomical location of the vertebral lesion at L5, between interiliac crests, a two-step surgery was mandatory. Three months after RT, the patient underwent the first surgical procedure, which consisted of a radicular decompression of the left L5 plus vertebral arthrodesis L3-S1. One month later, an L5 ventral corpectomy with vertebral stabilization with cementoplasty was performed. The histological study revealed the persistence of SEF as well as radiation-induced changes (Figures 11, 12). At two months follow-up, the patient presented with right sciatica, which was not present before surgery. Four months later, the patient described progressive mobility improvement but maintained dysesthesia at the right thigh; her spinal pain was controlled with tapentadol and gabapentin. The five-month post-operative MRI showed no persistence of the disease (Figures 13-19).

## Discussion

SEF is a rare mesenchymal tumor that occurs in middle-aged and elderly adults, most often arising in the upper or lower extremities or limb girdles, trunk, head, and neck. It is classified by the WHO as a malignant Fibroblastic/Myofibroblastic tumor, the same category as low-grade fibromyxoid sarcoma (LGFMS), malignant solitary fibrous tumor, fibrosarcoma, not otherwise specified (NOS), and epithelioid myxofibrosarcoma. Even though the spine is a rare primary site for SEF, the lumbar vertebrae are the most involved [1].

Some studies have described SEF as being part of the same spectrum as LGFMS, with SEF being considered pure SEF when only morphological characteristics of SEF are present and “hybrid SEF/LGFMS” when reminiscent areas of LGFMS are seen microscopically [8,11,12]. Immunohistochemical and molecular similarities have also been reported [8,11]. MUC4, currently used to diagnose SEF, is also positive in 99% to 100% of LGFMS cases [8]. Molecular studies revealed that the majority of LGFMS cases present FUS gene rearrangements, rare in pure SEF cases but present in all hybrid SEF/LGFMS cases as FUS-CREB3L2 gene fusion [8,11]. Pure SEF most commonly presents recurrent EWSR1 gene rearrangement, predominantly EWSR1-CREB3L1 and less frequently EWSR1-CREB3L2 [12]. Due to its rarity, SEF is frequently misdiagnosed, particularly when located in the bone [6]. Primary bone SEF can be confused with osteosarcoma due to its dense extracellular collagenous stroma that can mimic a malignant osteoid matrix. However, osteosarcoma typically presents a more prominent nuclear atypia, extensive matrix calcification with unequivocal lace-like neoplastic bone formation around individual cells, and strong nuclear positivity for SATB2; these characteristics can help in the differentiation from SEF. It can also be misdiagnosed as Ewing sarcoma because of its round and monomorphic appearance, even though sclerotic areas morphologically resembling SEF are rare. On immunohistochemistry, Ewing sarcoma presents strong membranous staining to CD99, while SEF may only demonstrate variably weak positivity [6,8]. Many authors also report that its epithelioid cell component in sclerotic hyalinized stroma often resembles sclerosing lymphoma, and others mention its resemblance to small cell sarcoma, not otherwise specified, and mesenchymal chondrosarcoma [8,12].

Furthermore, due to the rarity of this condition, the standard treatment for SEF still needs well-defined protocols. While a surgical approach is established, with many surgeons preferring a wide, en-bloc resection of the tumor, chemotherapy, and RT still lack specific evidence of efficacy. Thus, in the previous cases reported, RT has been used as either a neoadjuvant approach or an adjuvant one [3,13,14].

To our knowledge, 15 cases of primary spine SEF have been reported [1,3,4,13-15]. In 2004, Chow et al. published a case of SEF involving the left half of the sacrum, the left sacroiliac joint, and the posterior part of the left ilium. Six years after surgery, the patient presented with metastatic disease in the lung and scalp; two years later, the patient died of disseminated disease [15]. In 2018, two cases of SEF were reported: one with involvement of the posterior elements from C6 to T1 and infiltration of the T1 vertebral body, and another with a SEF located in the coccyx. Both were treated surgically, the first one with a two-stage posterior-anterior en bloc spondylectomy, followed by RT, and the second one with an en-bloc resection through the body of the S4 vertebrae [3,14]. In 2019, Liu et al. reported a case of SEF arising from the first lumbar vertebra that was treated with surgery (complete L1 vertebrectomy and laminectomy with T11-L2 pedicle screws and L1 cage fixation), followed by chemotherapy (four cycles of doxorubicin), with no signs of recurrence after 12 months [13]. Then, in 2020, a study of nine patients with SEF found two cases located on vertebrae (C6-T1 and L5-S1) with no information regarding metastasis [4]. Later in 2021, Righi et al. published a study that included six patients with SEF located along the spine and metastasis to the bones, lungs, and lymph nodes in three of them after 31-40 months of follow-up [1]. Finally, Tsuda et al. reported, in 2021, 21 cases of SEF in the bone, three of them in the spine (one in a thoracic vertebra, one in the cervical spine, and another in the sacrum), one of which presented with lung metastasis within 72 months of follow-up [6]. Thus, spine SEF has been described as having a tendency for local recurrence and distant metastasis, typically to the lung, bone, pleura, and brain, even after a few years [1,3,8]. Our case is still recent, having elapsed only 15 months since the diagnosis, so it is necessary to keep following up [8].

## Conclusions

In conclusion, SEF is an aggressive type of sarcoma that is easily misdiagnosed when presenting as a primary bone tumor. The need for its consideration in the approach to vertebral neoplasms and a correct diagnosis through morphology and immunohistochemistry is of the utmost importance to avoid unbeneficial treatments and detriment to patient survival. Moreover, further studies are necessary to clarify the efficacy of chemotherapy and RT in this context.
